# Patterns in sedentary and exercise behaviors and associations with overweight in 9–14-year-old boys and girls - a cross-sectional study.

**DOI:** 10.1186/1471-2458-7-16

**Published:** 2007-01-31

**Authors:** Saskia J te Velde, Ilse De Bourdeaudhuij, Inga Thorsdottir, Mette Rasmussen, Maria Hagströmer, Knut-Inge Klepp, Johannes Brug

**Affiliations:** 1 Erasmus University Medical Center Rotterdam, Department of Public Health, Rotterdam, The Netherlands; 2 Unit for Nutrition Research, Landspitali University Hospital, Reykjavik, Iceland; 3 Department of Social Medicine, University of Copenhagen, Copenhagen, Denmark; 4 Unit for Preventive Nutrition, Karolinska Institute, Stockholm, Sweden; 5 Department of Nutrition, Faculty of Medicine, University of Oslo, Oslo, Norway

## Abstract

**Background:**

Before starting interventions addressing energy-balance related behaviors, knowledge is needed about the prevalence of sedentary behaviors and low physical exercise, their interrelationships, possible gender differences. Therefore this study aimed to describe gender differences in sedentary and physical exercise behaviors and their association with overweight status in children from nine European countries. Additionally, to identify clusters of children sharing the same pattern regarding sedentary and physical exercise behavior and compare these groups regarding overweight status.

**Methods:**

Cross-sectional study among 11-year-old children in nine countries (n = 12538). Self-administered questionnaires assessed the time spent on TV viewing during dinner and during the day, PC use and on physical exercise. The parents reported children's weight and height. Descriptive statistics, cluster analyses, and logistic regression analyses were used for data analyses.

**Results:**

Boys spent more time on sedentary behaviors but also more on physical exercise than girls. High TV viewing and low exercise behavior independently increased the risk of being overweight. Based on the behaviors, five clusters were identified. Among boys, clear associations with being overweight were found, with the most unhealthy behavior pattern having the highest risks of being overweight. Among girls, high TV viewers and high PC users had increased risk of being overweight. In girls sedentary behaviors seemed more important than physical exercise with regard to overweight status.

**Conclusion:**

Despite selective non-response on BMI and reliance on self-reports, the associations between clusters and overweight in boys were clear, and differences between boys and girls regarding the behaviors and risks for overweight are noteworthy. These differences need to be considered when developing tailored intervention strategies for prevention of overweight.

## Background

Prevalence of childhood obesity is increasing in Europe as well as in other developed countries [1]. This is a major concern as it has harmful physical, psychological, behavioral, social and economic consequences. Overweight and obesity are results of a disturbed energy balance in which energy intake is higher than energy expenditure. During the past decades energy expenditure among children has decreased over time [2]. There are increased opportunities for children to be sedentary in their leisure time; especially TV viewing and electronic gaming are shown to be positively related with overweight [3-5]. Time spent on sedentary behaviors is inversely associated with physical exercise among adolescents [5,6], particularly among girls [4]; however, controversies exist on this issue [7].

Before starting interventions, knowledge is needed about the prevalence of sedentary behaviors and low physical exercise, their interrelationships, possible gender differences and their association with overweight. Large variations across countries in physical activity, time spent on TV viewing and PC use by children have been reported previously [8]. However, publications presenting gender differences regarding these prevalence rates, and gender differences in associations between overweight status and these behaviors, are scarce or not available [9]. Moreover, only a few studies explore the patterns between these different behaviors [9,10]. Usually only one or a small subset of individual health behaviors per study are examined. However, there is evidence that a combination of health behaviors may introduce a health risk that is greater than would be expected from the sum of the individual factors [11]. Instead of solely investigating associations between individual behaviors and overweight, exploring associations between health-behavior patterns and overweight can provide insights for the development of multifactorial overweight preventive strategies. Additionally, cluster analyses can identify subgroups that are at high risk for becoming overweight and might best benefit from interventions

The Pro Children Project [12] provides recent data concerning overweight status, TV viewing, PC use and physical exercise behavior and can therefore contribute to increase our knowledge in this area. The specific aims of the present study were therefore to: (I) describe the prevalence of sedentary and physical exercise behaviors; (II) assess how these behaviors are associated and whether specific clusters of behaviors can be identified; and (III) investigate whether the potential clusters are associated with overweight status, separately for boys and girls in nine European countries.

## Methods

### Sample

The Pro Children Cross-sectional survey (CSS) was designed to provide information on fruit and vegetable intake among European schoolchildren and on determinants of fruit and vegetable intake [12]. The methodology and sample has been described in detail previously [13,14]. Briefly, the survey was conducted in nine European countries (Austria, Belgium, Denmark, Iceland, the Netherlands, Norway, Portugal, Spain and Sweden) during October to December 2003. To obtain representative samples, schools were randomly selected from lists obtained from national or regional education authorities. Schools for special education were excluded from the sampling procedure. Participation rates among schools generally ranged from 70% to 100%, with Portugal (45%) and the Netherlands (30.4%) showing lower participation rates. For each country a minimum of 1300 eligible children were recruited. The recruited samples were nationally representative for each country, except for Austria and Belgium where the samples were drawn from regions (eastern Austria and Flanders respectively).

The general response rates were 90% and 76% for the child and parent questionnaires, respectively. The current study included the children that completed all questions on TV viewing, PC use and physical exercise (n = 12538, 94.2%). In this sample 49.9% were boys; mean age 11.4 years (8.8–13.8 years). More boys (6.2%) than girls (5.2%) did not respond to questions on all four behaviors (p = 0.002); no response differences according to age or parental reported family educational level were found.

Parental written informed consent was obtained prior to including the children in the survey. Research clearance was obtained from research ethics committees within all participating countries.

### Questionnaire and procedure

A comprehensive questionnaire was developed for both children and parents and translated and back translated to and from all relevant languages [15,16]. The children completed the questionnaire during a school hour in the presence of the class teacher who was asked to collect the data according to standardized procedures. The children took home a questionnaire to be completed by one of the parents. The retrieved data were entered according to standardized protocols at the national research centers. Thereafter, data files were submitted to the Pro Children Data Management Center at the University of Vienna where data processing and quality control took place. All protocols and questionnaires can be accessed at the Pro Children website [17].

### Measures

#### Behaviors

Usual TV viewing and PC use were measured by the questions 'About how many hours a day do you usually watch television and videos in your leisure time?' and 'About how many hours a day do you usually use a computer in leisure time?' with eight response alternatives, ranging from 'none at all' to '7 hours per day or more'. TV viewing during dinner was measured by a question with 5 response alternatives ranging from 'never' to 'every day'. The children's involvement in physical exercise was assessed by the question: 'how many hours per week do you usually exercise in your leisure time so much that you get out of breath or sweat?' with eight response alternatives, ranging from 'none at all' to '7 hours per week or more'.

Cut-off values were used to define the presence of sedentary behavior or low exercise behavior. According to recommendations [18], we used a cut-off of 2 hours/day for TV viewing. For TV viewing during dinner the cut off point was set at 'every day'. For PC use the cut off was set at > 1 hours/day, consistent with the cut-offs used by the HBSC study [8]. Low physical exercise behavior was defined as 0 – 3 hours/week involvement in leisure time physical exercise. This is different from the internationally well-accepted recommendation that children should engage in moderate-to-vigorous physical activities for at least one hour every day [19], because in the present study we were not able to assess engagement in physical activities during school hours or active transportation.

#### Test-retest reliability

Test-retest reliability regarding sedentary and exercise behavior was explored in a Dutch sample of 250 children with an average time period of 14.3 days (SD = 3.8). Using cut-off values as presented in this manuscript, more than 70% of the children were classified in the same group during test and re-test measurements (agreement ranging from 71.8% (PC use) to 86.7% (physical exercise)). No significant differences in the mean scores between test and retest for sedentary and exercise behavior were observed.

#### Body mass index (BMI) measurement and overweight classification

BMI was calculated as weight (kg) divided by height squared (m^2^) with the data reported by the parents (n = 7909, 63.1% of total). The response percentages were lowest for Portugal (56.4 %) and the highest for Belgium (73.8%). Non-responders were more likely to be boys (OR = 1.19 [1.11–1.29]), to watch TV during dinner every day (OR = 1.09 [1.00–1.17]), to report low physical exercise behavior (OR = 1.15 [1.07–1.25]) and report high PC use (OR = 1.10, [1.01–1.20]).

For international comparison age- and gender-specific child BMI cut-off points were used to define overweight (including obesity) [20].

Children's gender and exact age were retrieved from the child and parent questionnaires, respectively.

#### Family educational level

A potential confounder in the associations between the different behaviors and overweight status can be the economic position of the child's family, which was estimated by the family educational level. Family educational level was assessed in the parents' questionnaire. The highest level of education, reported by either parent was used as an estimate of the family educational level. In the analyses the variable was entered as a categorical variable: low educational level = less than 7 years of education, medium educational level = 7–9 years of education and high educational level = up to 12 years or university/college.

### Statistical analyses

Means, standard deviations and/or proportions were calculated for the key variables (using the mean value of an answer category when necessary).

Gender differences in proportions of the key behaviors were tested with Chi-square tests.

Pearson correlations were calculated between the different behaviors. K-means cluster analysis was used to identify specific patterns in the behaviors. K-means clustering places children in mutually exclusive groups characterized by their sedentary and physical exercise behaviors. Cluster analyses on all four behaviors were performed on standardized scores (z-scores) to overcome large impact from outliers and to provide a common metric for the solution. We specified four, five and six cluster solutions. The five-cluster solution was found to be adequate and meaningful regarding the different patterns found and is reported in the Results section. To test possible instability of the cluster solution, the solution was re-examined on a random sample of 50% of the total sample. The different clusters are given labels that characterize the behavioral pattern. These labels may reflect a value judgment, which was not intended as such, but was used as an efficient way to further describe results regarding the clusters.

The five clusters were then compared regarding the actual differences on the four behaviors by means of analyses of variance (ANOVA) and a Bonferoni post-hoc test. Logistic multilevel regression analyses were used to examine the risk of overweight associated with sedentary/physical exercise behaviors and with the five clusters. Multilevel analyses were used to take into account that the children were nested within schools and countries, and random effects by country were checked [21].

All analyses were performed separately for boys and girls and adjusted for age and additionally for family educational level. Cluster analyses were performed on the whole sample, while analyses on associations between clusters and overweight status were performed on a limited sample, due to missing data on BMI. Performing the cluster analyses on the whole sample instead of the limited sample was expected to give a more realistic reflection of the true patterns of the four behaviors, because missing data on BMI were selective and not random regarding the values on the four behaviors.

SPSS version 11.0.1 and MlwiN 2.01 [21] were used and the significance level was set at p < 0.05.

## Results

The prevalence of sedentary and low physical exercise behaviors varied by country (Table 1). TV viewing during dinner was least common in Norway and most often seen among Portuguese boys. All countries report averages higher than 2 hours/day of TV viewing for both genders, except for the Icelandic girls (1.7 ± 1.3 hours/day). Highest proportions of high TV viewing during the day were observed in the Netherlands and Belgium. High PC use was most common among Dutch boys and least common in Norwegian girls. In general, all sedentary behaviors were more common among boys than girls, with the largest differences in PC use. Girls more often reported low physical exercise behavior in all countries except for Denmark and Iceland.

**Table 1:** Gender-specific prevalence rates of the sedentary and low physical activity behaviors in nine European countries participating in the Pro Children study.

		TV during dinner Every day		TV viewing > 2 hours/day		Using PC > 1 hours/day		Physical exercise ≤ 3 hours/week	
Country	N	boy	girl	boy	girl	boy	girl	boy	girl

		%	%	%	%	**%**	%	%	**%**

Austria	1601	23.1	21.3	36.3	32.4	**40.9**	15.9	57.5	**72.4**
Belgium	1295	28.0	27.8	**50.1**	41.9	**35.4**	20.0	57.8	**74.7**
Denmark	1817	**18.9**	14.3	**37.6**	31.9	**39.0**	13.1	62.6	65.8
Iceland	1108	48.5	44.1	**35.2**	23.2	**35.7**	11.9	49.4	51.8
Netherlands	1089	**25.8**	21.1	50.2	45.5	**53.2**	26.1	55.0	**70.8**
Norway	1091	14.2	12.3	38.0	34.6	**23.9**	10.1	66.1	**77.5**
Portugal	2000	69.3	70.5	**49.0**	42.0	**39.8**	17.3	83.9	**94.9**
Spain	1223	**59.4**	52.3	**36.6**	31.4	**21.7**	15.3	63.9	**78.8**
Sweden	1314	21.3	22.9	31.6	31.0	**34.9**	17.5	57.0	**70.5**
Total	12538	**35.3**	33.4	**40.6**	35.3	**36.2**	16.4	62.7	**74.4**
Means (SD)		3.5 (3.0) d/week	3.3 (3.0) d/week	2.5 (1.7) h/day	2.2 (1.6) h/day	1.5 (1.6) h/day	0.84 (1.1) h/day	3.2 (2.3) h/week	2.5 (2.1) h/week

Numbers in **bold **indicate significant (p < 0.05) gender differences in prevalence

Overall, Portuguese boys were most likely to report sedentary behaviors as well as low physical exercise. Among girls, the Portuguese were also most likely to show these behaviors, except for high PC use. Swedish boys and Danish girls most often reported low sedentary and high physical exercise behavior.

### Clusters of sedentary and physical exercise behavior

Watching TV during dinner, TV viewing during the day and PC use were all statistically significantly positively correlated with each other (see Table 2), but not very strongly. Time spent on physical exercise was inversely correlated with TV viewing during dinner and during the day in girls, and with TV viewing during dinner in boys. Physical exercise was significantly positively associated with PC use in girls.

**Table 2:** Pearson's correlation coefficients between TV viewing during dinner, TV viewing, PC use and physical exercise behavior, separately for boys and girls participating in the Pro Children study.

N = 12538	TV dinner (days/week)	TV (hours/day)	PC use (hours/week)	Physical exercise (hours/week)
boys/girls	r	r	r	r

TV dinner	-	0.22***	0.10***	-0.14***
TV (hours/day)	0.25***	-	0.33***	-0.04**
PC use (hours/week)	0.14***	0.33***	-	0.05***
Physical exercise (hours/week)	-0.10***	-0.01	0.02	-

** p < 0.01 *** p < 0.001

The K-means cluster analyses resulted in five clusters for boys as well as girls. Three of the five clusters had more or less identical characteristics for both sexes. These three clusters were labeled as follows: Cluster 1: healthy behavior pattern; Cluster 2: high TV viewers; Cluster 4: high PC users;. Cluster 3 was characterized by low sedentary behavior & low physical exercise behavior in girls, but had a mixed pattern in boys. In boys Cluster 5 was labeled unhealthy behavior pattern because of the high scores on the sedentary behaviors and below average score on physical exercise. In girls, this Cluster was labeled 'high sedentary and high physical exercise', because of high scores on the sedentary behaviors as well as on the physical exercise behavior.

As can be seen in Table 3, the healthy cluster (1) is characterized by low z-scores (below 0) for TV during dinner, TV viewing and PC use combined with high (above 0) z-scores for physical exercise. However, the physical exercise score in the boys' cluster was only moderate (z-score = 0.10) and lower than the score for physical exercise in the boys' high TV cluster (z-score = 0.78). The high TV viewers (Cluster 2) are characterized by high values on TV viewing during dinner and during the day (z-scores > 0.76), but boys in this cluster were also highly physically active in contrast to girls in this cluster. The mixed group (cluster 3 in boys) had a high value for TV viewing during dinner (z-score = 0.99) but not for TV viewing during the day, and also low scores for PC use and physical exercise. In girls this cluster (Cluster 3: low sedentary and low physical exercise behavior) had low scores on all four behaviors. The high PC users (Cluster 4) are characterized by high z-scores on PC use (z-score > 1.47) but low to moderate z-scores on the other three behaviors. The unhealthy behavior cluster was characterized by high scores on the sedentary behaviors and low scores on physical exercise. The girls' high sedentary and high physical exercise cluster showed high scores on the sedentary behaviors but also above average scores on physical exercise (z-score = 0.28).

**Table 3:** Cluster centers in z-scores for the four behaviors included in the cluster analyses, prevalence of overweight and adjusted risk for overweight, separately for boys and girls participating in the Pro Children study.

Cluster	Number (%)	TV during dinner	TV viewing	PC use	Physical exercise	Overweight (%) (95% CI)	Adjusted risk for overweight (Boys, n = 3809; girls, n = 4100)	
Boys							OR	95% CI

1	2624 (42.0%)	-0.92^a^	-0.43^a^	-0.44^a^	0.10^a^	12.7 (11.1–14.3)	1	
2	1100 (17.6%)	0.79^b^	0.76^b^	-0.25^b^	0.78^b^	17.1 (14.2–19.9)	1.25	0.97–1.62
3	1494 (23.9%)	0.99^c^	-0.28^c^	-0.30^b^	-0.73^c^	21.7 (19.0–24.5)	**1.50**	1.18–1.90
4	601 (9.6)	-0.48^d^	-0.03^d^	1.47^c^	0.01^a,d^	17.9 (13.9–21.9)	**1.43**	1.05 – 1.96
5	436 (7.0%)	0.83^e^	1.68^e^	2.26^d^	-0.10^d^	22.2 (17.0–27.4)	**1.67**	1.18 – 2.37

Girls								

1	1337 (21.3%)	-0.34^a^	-0.30^a^	-0.26^a^	1.55^a^	11.4 (9.3–13.5)	1	
2	1339 (21.3%)	0.95^b^	0.95^b^	-0.24^a^	-0.41^b^	20.0 (17.3–22.7)	**1.64**	1.23 – 2.18
3	2794 (44.5%)	-0.38^a^	-0.49^c^	-0.36^b^	-0.47^b^	12.6 (11.1–14.2)	1.03	0.80 – 1.33
4	584 (9.3%)	0.15^c^	0.17^d^	1.57^c^	-0.18^c^	16.0 (12.4–19.7)	**1.42**	1.00 – 2.01
5	229 (3.6%)	0.58^d^	2.01^e^	3.23^d^	0.28^d^	15.4 (9.6–21.3)	1.33	0.80 – 2.21

a,b,c,d,e for significant difference (p < 0.05) between clusters with post-hoc Bonferoni testing

* ORs are adjusted for age; random intercept was allowed on country and school level

Being in the healthy behavior cluster discriminated very well for everyday TV viewing during dinner (nobody exhibits this behavior) and for low physical exercise in girls (nobody reported physical exercise < 4 hours/week) (data not shown). The unhealthy cluster discriminated very well for high TV viewing during the day (98.2% for boys; 93.9% for girls) and high PC use (100% for boys and girls)

The clusters including most children were the healthy behavior cluster in boys (42%) and the low sedentary and low physical exercise cluster in girls (44.5%). The unhealthy behavior clusters included 7.0% of the boys and about 3.6% of the girls.

### Associations with overweight

The highest prevalence of overweight was found in Portugal (21.6% for boys and 19.5% for girls) and Spain (17.9% for boys and 18.4% for girls). Lowest prevalence values for overweight were found among Belgian boys (8.3%) and Dutch girls (7.9%).

High TV viewing during dinner and during the day, and low physical exercise behavior (in boys only) was associated with a risk of being overweight (see Table 4). High PC use was not associated with being overweight in boys or girls. These findings did not change after adjustments for family educational level (data not shown).

**Table 4:** Odds ratios (OR) and confidence intervals (CI) for being overweight (including obesity) for TV viewing during dinner, TV viewing, PC use and physical exercise behavior (adjusted for each other), separately for boys and girls participating in the Pro Children study.

	Boys (n = 3809)			Girls (n = 4100)		
Behavior	OR*	95% CI		OR*	95% CI	

TV during dinner every day	1.31	1.07	1.59	1.30	1.06	1.59
TV viewing > 2 hours/day	1.33	1.11	1.61	1.30	1.07	1.58
PC use > 1 hours/day	1.15	0.95	1.39	1.09	0.86	1.39
Physical exercise ≤ 3 hours/week	1.57	1.29	1.90	1.17	0.94	1.46

* ORs are adjusted for each other and for age; random intercept was allowed on country and school level

The healthy behavior cluster had the lowest proportions of overweight boys (12.7%, (11.1–14.3%)) and girls (11.4%, (9.3–13.5%)). In girls, the cluster with low sedentary and low physical exercise behavior had the second lowest proportion of overweight girls (12.6%, (11.1–14.2%)) (Table 3).

In boys three clusters had a significantly increased risk of being overweight (Table 3). Only the high TV viewers, who also had high scores on physical exercise, did not significantly differ in risk of being overweight from the boys in the healthy behavior cluster. In girls, the high TV viewers and the high PC users had the highest proportions of overweight, and significantly increased risks of being overweight. The odds ratios (ORs) for the low sedentary and low physical exercise cluster and the high sedentary and high physical exercise cluster were also >1, but not significant. The estimated ORs were not influenced by adjustments for family educational level (data not shown)

## Discussion

This international study showed that the prevalence of sedentary and physical exercise behavior varies across countries and by gender, with boys exhibiting more typical recreational sedentary behaviors but also being more physically active than girls. Moreover, these behaviors, except PC use, were associated with overweight status. Furthermore, among boys the unhealthy behavior cluster had the highest risks of being overweight.

The pronounced differences between boys and girls in sedentary and physical exercise behaviors were observed across all countries and have been reported by others [9,22-24]. However, most studies to date have data only on either sedentary or physical activity behavior, of which some report gender differences and others not [3,24,25]. Moreover, as with the current study, often only typical sedentary behaviors were assessed, such as TV and video viewing and PC use, while Jago et al. [26] showed that girls engage in activities that are often not assessed, for instance personal care and social interactions. The gender difference in physical activity seems more consistent in the literature [24,27-29], while contradictory results have been reported regarding TV viewing [24,25,29,30].

Notable in the present results are the high proportions of low physical exercise behavior among girls. This may be an overestimation caused by the way the question was formulated and the typical physical activities in which girls normally engage. Children can be active in many different ways. Boys may be more often enrolled in organized sports and competitive, team, and high intensity sports while girls spend time on unorganized, non-competitive and medium to low intensity sports and exercises [9,28,29,31], which were not assessed asked for in the questionnaire. Nevertheless, the HBSC study used a more extensive questionnaire and a cut-off of ≥ 5 days/week of 60 minutes of vigorous physical activity, and their results do not differ greatly from ours [8]. Adjusting the cut-off in the present study to < 2 hours/week, still results in 76.1% of the Portuguese girls being characterized as low physically active. A study conducted in Porto, Portugal [32] also reported a high proportion of inactive girls (71.7%) and the HBSC study reported that Portugal was consistently in the bottom quartile regarding physical activity in any age group [23]. Prevalences of the other behaviors and proportions of overweight in the current study is in line with observations from the HBSC study [8,23].

Associations between sedentary behavior and physical exercise are subject to debate [7,9,33]. The 'displacement hypothesis' proposes that sedentary behavior displaces physical activity; however several studies, including ours, only find weak or no associations [9,24,34]. In line with this, we found that boys in the high TV viewers cluster had above average physical exercise levels, and the low sedentary and low physical exercise cluster in girls indicates that a large proportion of girls spends little time on TV viewing and PC use as well as little time on physical exercise. Likewise a small part of the girls sample combines high sedentary behavior with above average involvement in physical exercise (i.e. the high sedentary and high physical exercise cluster). This latter finding supports the idea that some sedentary behaviors and physical activity can co-exist. Physical activity may be executed typically in the afternoon while sedentary behaviours are more prevalent later at night [7]. The multifaceted character of sedentary behavior and the fact that we measured only a selection of possible sedentary behaviors may have caused the apparently conflicting.

We found that among girls the high TV viewers and the high PC users had an increased risk of being overweight. That TV viewing is positively associated with BMI or overweight status in girls is in line with other studies [3,24]. Both clusters also had high proportions of girls reporting low physical exercise (≥ 85%) and almost half of the high PC users spent more than 2 hours/day on TV viewing (data not shown). The main difference between these two clusters and the low sedentary and low physical exercise cluster is the time spent on TV viewing and PC use. Since the low sedentary & low physical exercise cluster did not show an increased risk of being overweight, it appears that for girls sedentary behavior is a more important factor with regard to overweight status than is physical exercise. This hypothesis is also supported by the observation that the high sedentary and high physical exercise cluster showed increased odds (OR>1), although not statistically significant, for being overweight and the proportion of overweight girls in this cluster was the second highest. Absence of statistical significance may be due to a low number of girls in this cluster.

In boys, all clusters had increased risks of being overweight compared with the healthiest behavior cluster, except the high TV viewers. This latter cluster also had the lowest proportion of boys reporting low physical exercise and since physical exercise was associated with overweight status, this may explain the absence of elevated risk. So, in contrast to girls, in boys both sedentary behavior and physical exercise seem to be important with regard to overweight.

It can be argued that the variable TV viewing during dinner is not a real indicator of sedentary behavior and should thus not be included in the present analyses. However, we included this variable, because it can provide new information. Since this variable has not been included in this line of exploration of specific energy-balance behaviors before, since it has been argued that eating while watching television may be one of the reasons for the positive association between TV viewing and overweight, and because in weight management programs it is advised to not engage in distracting activities such as TV viewing while eating. It can be argued that the variable 'TV during dinner' may be associated with weight management and thus overweight. Analyses of the data without this variable, resulted in the same conclusions for boys, but showed a less clear pattern in girls (data not shown). Although, again, in girls TV viewing behavior appeared to be more important than physical exercise behavior with regard to overweight status, which is in line with the present results.

Adjustment for family educational level did not change the estimated ORs for being overweight, indicating that family educational level did not confound these associations. This can probably be explained by the fact that in the multilevel logistic regression analyses results were already adjusted for country, and that the different educational levels were not equally distributed over the nine countries. Spain and Portugal showed higher proportions of lower educational levels, and also showed higher levels of overweight.

Some limitations of the present study should be addressed. Since we solely rely on cross-sectional data, we cannot draw conclusions about causal relations. Children who are highly sedentary might become overweight, or overweight children might become more sedentary. Secondly, we used self-reported data for the behaviors, which is less accurate than observed measures. If children tend to underestimate their time spent on sedentary behaviors, the prevalence of these behaviors would be higher than presented here. If underestimation is more common among overweight children, the observed effect estimates would be higher if more accurate measures were used. However, Schmitz et al. [35] showed that a brief self-administered questionnaire is as good for group comparisons as a detailed seven-day logbook regarding TV viewing and PC use.

Thirdly, we used parent-reported data to calculate children's BMI. In general people tend to overestimate their height and underestimate their weight, resulting in an underestimation of BMI [36], which is also more common when parental reporting is used [37,38]. A Norwegian study validated a methodology very similar to the Pro Children survey but using self-report by children, completed at home [39]. In that study, there was a relatively good correspondence between reported and measured height and weight in children of a comparable age to that in our study.

The non-response on BMI was different between the clusters for boys, with the highest rates in the unhealthy behavior cluster (43.1%) and the mixed cluster (42.7%) and lowest in the healthy behavior cluster (36.2%). This suggests that an even clearer pattern would probably have emerged without this selective non-response. The non-response rates on BMI were lower in girls, and also varied less between the clusters (32.7% – 36.6%, not significant).

Besides these limitations, a strength of this study is that it provides representative information from a large sample from nine countries across Europe. A special feature of this study was the cluster analyses, investigating associations with overweight between patterns of behaviors instead of individual behaviors. In our analyses we also took into account that children were nested within countries and observed no random effects by country (data not shown). So, despite the geographical, socio-economic, cultural and climate differences, the associations shown are consistent across all countries.

## Conclusion

We can conclude that the prevalence of sedentary and low physical exercise behavior varies across countries and by gender. Boys appeared to engage in more sedentary behavior, while girls participated in less sedentary behavior but were also less physically active. In boys, both sedentary and physical exercise appear to be important with regard to overweight, while in girls the typical sedentary behaviors (TV viewing, PC use) seem more important than physical exercise behaviors in the association with overweight. These differences between boys and girls are important to consider when developing tailored intervention strategies for prevention of overweight.

## Competing interests

The author(s) declare that they have no competing interests.

## Authors' contributions

SJtV carried out the analyses and drafted the manuscript; IdB participated in the design of the study, supported in the statistical analyses and helped to draft the manuscript; IT participated in the design of the study and helped to draft the manuscript; MR helped to draft the manuscript; MH helped to draft the manuscript; KIK developed and directed the overall study and helped to draft the manuscript; JB developed and directed the overall study and coordination and helped to draft the manuscript. All authors read and approved the final manuscript.

## Pre-publication history

The pre-publication history for this paper can be accessed here:


